# Gastroblastoma Treated by Endoscopic Submucosal Excavation with a Novel *PTCH1::GLI2* Fusion: A Rare Case Report and Literature Review

**DOI:** 10.3390/curroncol29110697

**Published:** 2022-11-17

**Authors:** Yongru Liu, Huanwen Wu, Xi Wu, Yunlu Feng, Qingwei Jiang, Qiang Wang, Aiming Yang

**Affiliations:** 1 State Key Laboratory of Complex Severe and Rare Diseases, Department of Gastroenterology, Peking Union Medical College Hospital, Peking Union Medical College and Chinese Academy of Medical Sciences, Beijing 100730, China; 2 Department of Pathology, Peking Union Medical College Hospital, Peking Union Medical College and Chinese Academy of Medical Sciences, Beijing 100730, China

**Keywords:** gastroblastoma, stomach tumor, *PTCH1::GLI2* fusion, *MALAT1-GLI1* fusion, endoscopic submucosal excavation, endoscopic treatment

## Abstract

Gastroblastoma is an extremely rare stomach tumor that primarily presents in adolescent and early adulthood, with a biphasic cell morphology of epithelioid and spindle cells. In light of its similarity to other childhood blastomas, it has been named gastroblastoma. Few patients showed a potential of metastasis and recurrence, however, most of the reported cases were alive, with no evidence of the disease after surgical treatment. Commonly, *MALAT1-GLI1* fusion has been considered to be the most relevant mutation. Herein, we present a case of an asymptomatic 58-year-old man who happened to find a submucosal gastric mass during a gastroscope and received endoscopic submucosal excavation (ESE). He turned out to have a gastroblastoma with a novel *PTCH1::GLI2* fusion confirmed by Sanger sequencing. The patient was discharged two days after ESE without any complication and was recurrence-free during his one-year follow-up. According to the previous literature and our own experience, in cases with characteristic histopathology and immunohistochemistry patterns, a diagnosis of gastroblastoma should be considered even without a *MALAT1-GLI1* fusion. Gastroblastoma pursues a favorable clinical outcome and endoscopic therapy could be an effective alternative treatment choice.

## 1. Introduction

Gastroblastoma, an extremely rare stomach tumor, is characterized by biphasic morphology with nests, sheets, tubules and cords of bland epithelioid cells, as well as clusters of spindle cells [1]. It usually lacks typical symptoms and may predominantly occur in younger patients with an onset age ranging between 9 and 74 years old (with a median age of 28 years old) and there is no apparent gender predilection. Most commonly seen in the antrum of the stomach, gastroblastoma often occurs in the gastric wall and inconstantly involves the submucosa, mucosa and subserosa, and may occasionally be transmural and give rise to peritoneal or lymph node metastases [2]. First described in 2009 by Miettinen et al. [3], gastroblastoma was first included in the fifth edition of the World Health Organization (WHO) classification of tumors of the digestive system in 2019. Due to its rarity, the etiopathogenesis and molecular alterations have been largely unknown, and consensus on the appropriate treatment has not been reached. Hitherto, there were only 15 cases being reported and *MALAT1-GLI1* fusion was considered to be the most relevant mutation [4]. However, there was a lack of description of treatment for one patient, and all the other 14 patients took surgical treatment and most of them (79%, 11/14) had a good prognosis, without evidence of metastasis or recurrence. Herein, we report a gastroblastoma with a novel *PTCH1::GLI2* fusion in a 58-year-old male who successfully underwent endoscopic submucosal excavation (ESE) with no metastasis or recurrence post-procedure during his one-year follow-up, and summarize the relevant literature. We hope this will enlighten the diagnosis and treatment choices for gastroblastoma patients in the future.

## 2. Case Presentation

**Figure 1:**
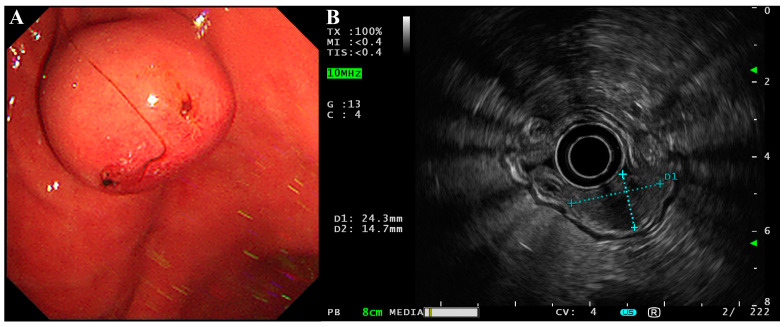
EGD and EUS of the gastroblastoma. (**A**) EGD showed a submucosal mass with a small ulcer on its surface at the lesser curvature of the gastric body. (**B**) EUS showed the lesion was hypoechoic with a clear boundary and uniform internal echo. The lesion was approximately 2.43 × 1.47 cm in size and originated from the muscularis propria with an intact serosa layer.

The patient was a 58-year-old male who happened to find a submucosal mass during an esophagogastroduodenoscopy (EGD) in an annual routine examination. He had no discomfort or history other than hypertension and cholecystlithiasis, and came to our hospital for further consultation and examination. We found the mass at the lesser curvature of the gastric body (2.43 × 1.47 cm, Figure 1A) with an ulceration in the center of the mucosal surface. Endoscopic ultrasonography (EUS) was then performed using a circular array (EU-ME2 PREMIER PLUS with UE260-AL5, Olympus Optical Co., Ltd., Tokyo, Japan). The mass was hypoechoic and originated from the muscularis propria with a clear boundary and uniform internal echo (Figure 1B). There was no sign of infiltrative growth as the serosa layer of the lesion was intact. It was initially thought to be a gastrointestinal stromal tumor (GIST).

**Figure 2:**
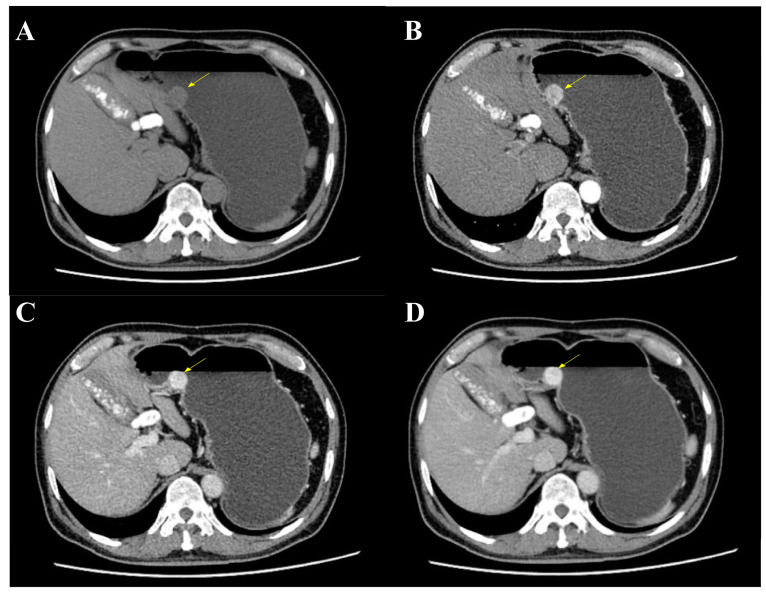
Abdominal and pelvic enhanced CT. (**A**) CT showed a mass at the gastric body with a clear boundary and soft tissue density (yellow arrow). (**B**) The mass was enhanced during arterial phase (yellow arrow). (**C**) An increased enhancement of the mass was found during portal venous phase with a maximum CT value of 156HU (yellow arrow). (**D**) The mass was still enhanced during delayed phase (yellow arrow).

The mass was also revealed during an abdominal and pelvic enhanced computer tomography (CT), with a soft tissue density and a clear boundary (Figure 2A). It showed a significant enhancement during the arterial phase, portal venous phase and delayed phase (Figure 2B–D). A significantly increased enhancement of the mass was found during the portal venous phase, with a CT value of 156HU, and a glomangioma was suspected. With the exception of multiple gallstones, no lymph nodes or other abnormal phenomenon were found.

**Figure 3:**
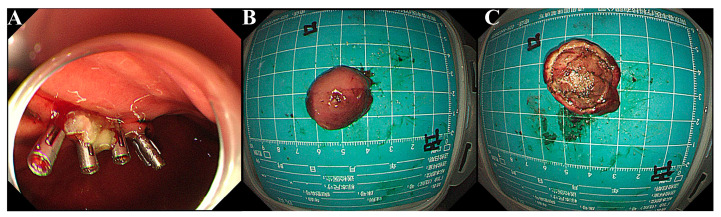
ESE and gross features of the mass. (**A**) After ESE’s en bloc excavation, four clips were used to close the incision. (**B**,**C**). Grossly, the mass was yellowish pink with a pink-gray cut surface.

Physical examinations and laboratory tests of the patient showed no abnormalities. As his lesion had a clear boundary without infiltrative growth, the patient was admitted to our hospital for ESE using a GIF-Q260J with a LUCERA CV-290 electronic endoscope system (Olympus Optical Co., Ltd., Tokyo, Japan). ESE was successfully carried out and four clips were used to close the wound after complete excavation on 26 July 2021 (Figure 3A). It had a small ulcer on the surface and was solid, yellowish-pink with a pink-gray cut surface (Figure 3B,C).

**Figure 4:**
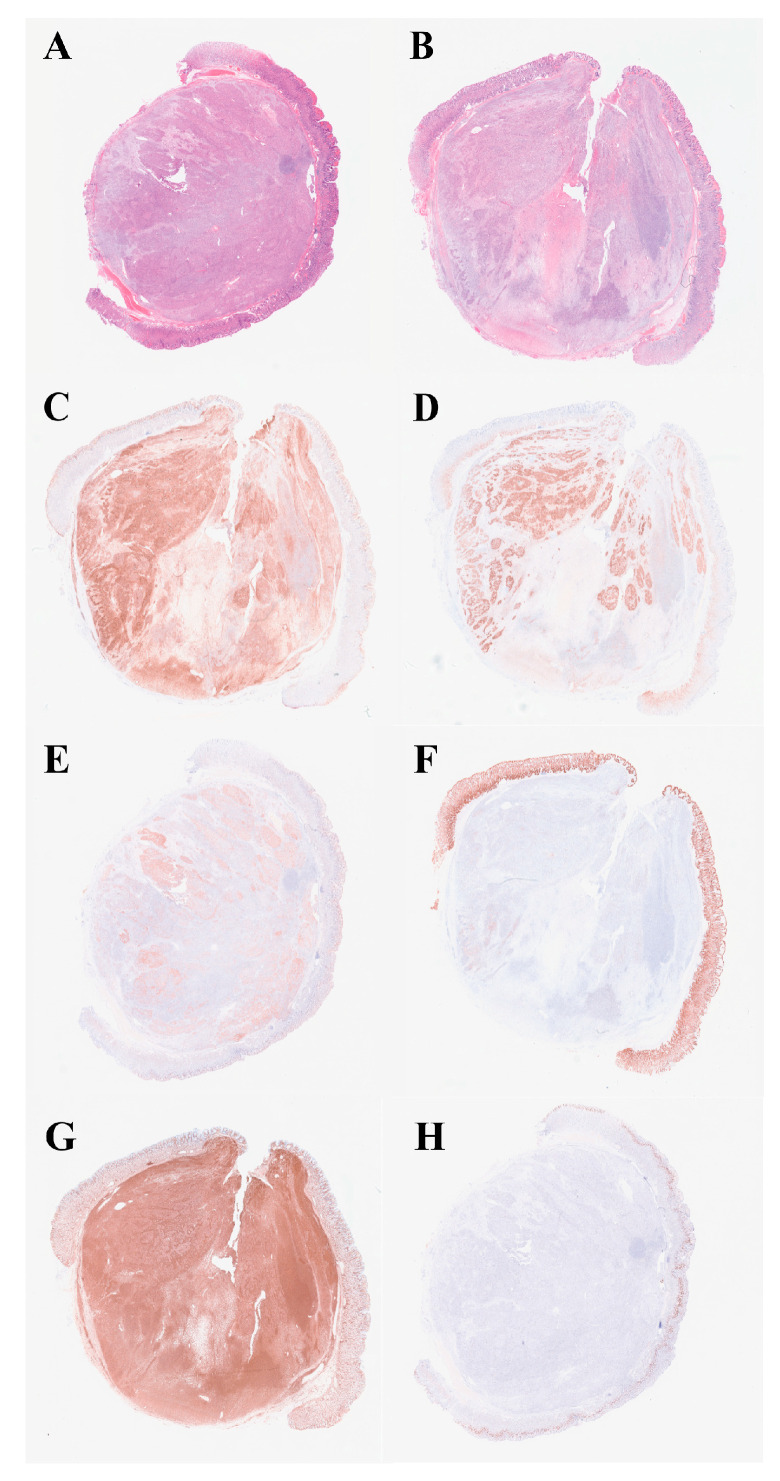
Hematoxylin-eosin staining (H&E) and IHC profile. (**A**,**B**) Histologically, the lesion had a biphasic pattern with spindle cells and epithelioid cells (in different layer, H&E, ×40). (**C**) CD10 was positive in both components (IHC, ×40). (**D**) CD56 was positive in the epithelioid part (IHC, ×40). (**E**) S100 was expressed in the epithelioid component (IHC, ×40). (**F**) EMA was focally expressed in the epithelioid part (IHC, ×40). (**G**) Vimentin was positive in both components (IHC, ×40). (**H**) Ki-67 index was approximately 5% (IHC, ×40).

**Figure 5:**
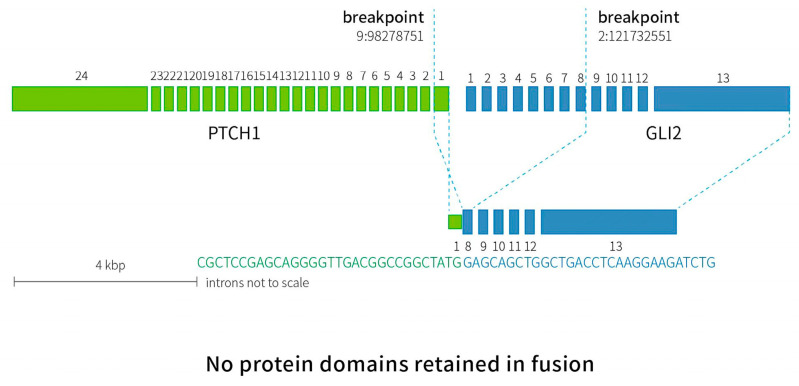
A diagram of RNA-based NGS results confirmed by Sanger sequencing. Inter-chromosomal fusion was identified between exon 1 of *PTCH1* and exon 8 of *GLI2*. Breakpoint confirmed by Sanger sequencing was indicated by dotted lines (*PTCH1* chr9:98278751, *GLI2* chr2:121732551).

Histologic examination showed a biphasic pattern with relatively uniform spindle cells and epithelioid cells (Figure 4A,B, from different cut layer). As for immunohistochemistry (IHC), the lesion was positive for CD10 and vimentin in both the spindle cells and the epithelioid cells, while positive staining of CD56, S100 and EMA were mainly found in the epithelioid part (Figure 4C–G). Moreover, CD34, CD117, DOG-1, SMA, desmin, AE1/AE3, CAM5.2, CgA and Syn were negative. The Ki-67 index was approximately 5% (Figure 4H). Specific pathological details have been reported by our pathologist [5]. RNA was extracted from formalin-fixed paraffin-embedded (FFPE) specimens and RNA-based NGS was performed. A novel inter-chromosomal fusion, occurring between *PTCH1* and *GLI2* (ex1:ex8), was revealed. The fusion was subsequently confirmed by a reverse transcriptase polymerase chain reaction (RT-PCR), followed by Sanger sequencing (Figure 5). Based on the above evidence, the patient was diagnosed with gastroblastoma. The patient recovered well without any complication and was discharged two days after the ESE procedure. During the one-year follow-up, with two EGD re-examinations and one CT scan, the patient had no evidence of recurrence or metastasis. Close follow-up was recommended.

## 3. Discussion

Until the present case, there were only 12 articles, reporting 15 gastroblastoma cases. Patients usually presented with atypical symptoms including abdominal pain or melena. Various imaging examination showed that gastrbolastoma was usually hypoechogenic in EUS and with cystic components or uneven enhancement in CT. The detailed basic clinical and histological information is summarized in Table 1 and Table 2. All but one of the patients had surgery, with multiple options including tumorectomy, partial or subtotal gastrectomy by laparotomy, or laparoscopy based on the tumor size, location, infiltration and metastasis condition. Two patients also received extra treatment: one received chemotherapy before the surgery but had no response; the other received postoperative radiation. The clinical follow-up varied across different patients, with most of them (79%, 11/14) having no evidence of metastasis or recurrence (Table 3). Our patient was the only one treated with endoscopic therapy, and still had no metastasis or recurrence in the one-year follow-up following the procedure. Miettinen et al. proposed that gastroblastomas were lymphophilic tumors and indicated the importance of lymph node dissection [3]. However, these surgeries can cause extensive trauma with high risks and a long recovery time. As most of the gastroblastomas behave indolently, and considering most reported cases, as well as our case, had an excellent prognosis, we hope this endoscopic therapy will enlighten the future treatment of gastroblastoma patients, particularly for those who have small foci restricted to the submucosa without invasive growth.

Gastroblastoma is solid and sometimes contains cystic hemorrhage. Histologically, the tumor has a biphasic epithelioid and spindle cell morphology. As its histomorphologic characteristic is similar to that of some blastomas occurring in other organ (especially pleuropulmonary blastoma and nephroblastoma), it was named after gastroblastoma by Miettinen et al. [3]. When comparing gastroblastomas to other blastomas which have immature components and definite malignant potentials, gastroblastomas tend to demonstrate an excellent prognosis, probably due to its developed architecture and low-grade cytologic features. It has been proposed that gastroblastoma may be better related to the spindle epithelioma of thymus and the demyelinating nested spindle cell tumor of liver rather than blastomas [2]. Due to its biphasic morphology, it is almost impossible to diagnose gastroblastoma by biopsy specimens. Its spindle components can be misdiagnosed as a low-grade sarcoma, the solid nested region as a neuroendocrine tumor, and the tubuloid structure as a form of epithelial neoplasm. By IHC, gastroblastoma seldom expresses markers that are usually positive in GISTs (c-KIT or CD117 and DOG-1), solitary fibrous tumors (CD34), nerve sheath tumors (S100), or mesothelial tumors (calretinin, CK5/6, WT-1). Neuroendocrine tumor markers (CgA and Syn) and smooth muscle tumor markers (SMA and desmin) are not usually expressed in gastroblastoma. Further main points of differential diagnosis are listed in Table 4. Notably, our case was positive for S100, which is not common in gastroblastomas. Nonetheless, it had a characteristic biphasic morphology, and its histopathology pattern and IHC features were relatively consistence with previous reports, which helped us reach the final diagnosis of gastroblatoma. As for a definite diagnosis of gastroblastoma, comprehensive evaluation of clinical and pathological features, including tumor origin, histomorphology, IHC patterns and mutation, should be considered.

A recurrent *MALAT1-GLI1* fusion has been detected in five cases of gastroblastoma (5/15 cases). In addition, it can also be involved in plexiform fibromyxomas (3/16 cases) [15]. Graham et al. summarized in their study that a reciprocal *GLI1-MALAT1* fusion was found in two gastroblastoma cases and for the other two cases with a *MALAT1-GLI1* fusion, MALAT1 exon1 was fused to *GLI1* intron 5 to exon 12 and *GLI1* exons 5–12, respectively [4]. *MALAT1* (metastasis-associated lung adenocarcinoma transcript 1) is a long noncoding RNA, which is highly expressed in the nucleus of most cells and provides a strong promoter that drives *GLI1* overexpression [16]. *GLI1* (glioma-associated oncogene homologue 1) encodes a zinc-finger transcription factor in the Kruppel family proteins, and plays a vital role in the Sonic Hedgehog (Shh) pathway, as well as in proliferation, growth, angioinvasion, invasion, migration and cancer stem cell self-renewal in a variety of malignant tumors [17]. *MALAT1-GLI1* fusion results in the overexpression of *GLI1* and, in turn, the overexpression of *GLI1* leads to the overexpression of several downstream targets, including *PTCH1*, *VEGFA*, *SOX2* and *CCND1*. *PTCH1* (transmembrane receptor Patched 1) is involved in cell proliferation and organization, and it is correlated with a copy number variation burden, tumor mutational burden (TMB) and loss of heterozygosity score [4]. *PTCH1* and *GLI* family proteins are both involved in the Hedgehog-GLI (hh-GLI) pathway. Canonical activation of hh-GLI signaling is initiated when the secreted ligand binds to *PTCH1*, which derepresses SMO (G protein-couple receptor Smoothened) and leads to the activation of the GLI transcription factors (*GLI1*, 2, 3) [18]. Abnormal activation of hh-GLI has been implicated in a wide variety of tumors, such as those of the skin, brain, lungs, gastrointestinal, pancreas, prostate, breasts and ovaries [19]. Kim etal reported a recurrent *FHL2-GLI2* fusion in a sclerosing stromal tumor, which resulted in the transcriptomic activation of the Shh pathway [20]. Punjabi et al. presented a high-grade uterine sarcoma case with a *PAMR1::GLI1* fusion in which the Shh/GLI signaling pathway was involved [21]. A translocated *ACTB-GLI1* fusion was reported by Ambrosio et al. in a patient with a malignant epithelioid neoplasm of the ileum [22]. The ubiquitously expressed *ACTB* activated *GLI1* expression, where the Shh signaling pathway was involved in the tumorigenesis. As for our case, the gastroblastoma patient with a *PTCH1::GLI2* fusion, we predicted that the Shh pathway was activated and an overexpression of *GLI2* would be involved, similar to that of other GLI fusion tumors. The underlying mechanism and downstream targets require further study.

## 4. Conclusions

We report a gastroblastoma occurring in an adult with a novel *PTCH1::GLI2* fusion. In cases with a characteristic histopathology and IHC patterns, a diagnosis of gastroblastoma should be considered, even without a *MALAT1-GLI1* fusion. For this particular case, whose lesion was relatively small with a restricted growth, a therapeutic endoscopy was carried out and no evidence of metastasis or recurrence was indicated during the one-year follow-up. Combining the previous studies, it seems that this particular tumor possesses a favorable clinical outcome and prognosis, despite its adopted suffix “blastoma”. Additionally, endoscopic therapy could be an effective alternative choice for some gastroblastoma patients in the future.

## Figures and Tables

**Table 1:** Clinical Features of Reported Gastroblastomas.

Author	Time	Age	Country	Sex	Main Complaint	Images
Selene C. Koo [6]	2021	17	USA	M	3 days of bright red hematemesis and melena	EGD showed a large ulcerated sessile submucosal gastric mass. (No further detail has been described)
Long Weiguo [7]	2020	53	China	F	1 year of epigastric pain and discomfort	CT showed a lobulated mass in the antrum with exophytic growth towards the lumen, with clear borders and uneven enhancement.
Diogo Nogueira Pinto [8]	2019	53	Portugal	F	Heartburn and dyspepsia	UDE showed a submucosal lesion. EUS showed the lesion was hypoechogenic with irregular border, cystic components and calcifications.
Federica Castri [9]	2019	79	Italy	M	Weight loss and dysphagia	Contrast-enhanced CT showed an antral transmural thickening. EUS showed a submucosal hypoecogenic mass.
Giovanni Centonze [10]	2019	43	Italy	F	bleeding	Endoscope revealed a submucosal, ulcerated lesion and it showed inhomogeneous enhancement in EUS and CT.
Omar Toumi [11]	2017	29	Tunisia	F	8 months of epigastric pain and 2 days of hematemesis	Upper gastrointestinal endoscopy revealed a submucosal lesion. CT scan showed the tumor was solid-cystic.
Rondell P Graham [4]	2017	27	USA	M	Not mentioned	Not mentioned.
		56		F	Not mentioned	Not mentioned.
Ma Yangyang [12]	2014	12	China	M	More than 3 months of intermittent blood in stool and abdominal mass	CT and MRI revealed a mass in the gastric antrum, and gastroscope showed the mass had an ulcer approximately 2 × 1 cm.
Teresa Fernandes [13]	2014	19	Portugal	F	5 months of diffuse abdominal pain and a mass in the right quadrants of the abdomen	Abdominal ultrasonography revealed an anechoic well-defined complex cystic lesion. CT showed the mass was lobulated with some foci of punctiform calcification. The wall, internal septa and adenopathy demonstrated moderate enhancement after administration of intravenous contrast material. MRI showed the fluid content of the loculi was homogeneously hypointense on T1-weighted images and markedly hyperintense on T2-weighted images.
Elizabeth A. Wey [2]	2012	28	USA	M	Constipation after a motor vehicle accident	CT revealed a heterogeneous mass in the gastric antrum. (No further detail has been described.)
Dong Hoon Shin [14]	2010	9	Korea	M	3 months of abdominal pain and a periumbilical mass	CT revealed a solid and cystic gastric antral mass which compressed the adjacent duodenum, gallbladder and pancreas.
Markku Miettinen [3]	2009	19	USA	M	Nonspecific abdominal pain	Not mentioned.
		27		F	Nonspecific abdominal pain	Not mentioned.
		30		M	Anemia, fatigue	Not mentioned.

Notes: Time refers to the publication time. Age was measured by years old. Abbreviations: M, male; F, female; EGD, esophagogastroduodenoscopy; CT, computerized tomography; UDE, upper digestive endoscopy; EUS, endoscopic ultrasonography; MRI, magnetic resonance imaging.

**Table 2:** Mutation and IHC patterns of gastroblastoma.

Author	Mutation	IHC (+)	IHC (−)
Selene C. Koo [6]	EWSR1-CTBP1 fusion and overexpressed NOTCH and FGFR	Vimentin, CD56, CD10, PCK and Syn, Ki-67 (5%)	CD34, DOG1, CD117, S100, desmin, CgA
Long Weiguo [7]	Not mentioned	PCK ^e^, vimentin ^s^, CD10, CD56, Ki-67 (<2%)	SMA, calponin, calretinin, CD117, CD34, DOG1, CK5/6, CK7, CK20, S100, CgA, SDHB
Diogo Nogueira Pinto [8]	Not mentioned	Vimentin ^s^, CD10, CD56, MNF ^e^, CAM5.2 ^e^, AE1/AE3 ^e^, Ki-67 (<5%)	CD34, CD117, desmin, caldesmon, HMB45, WT1, Syn
Federica Castri [9]	*MALAT1-GLI1* fusion	Vimentin ^s^, CD10, cytokeratins AE1/AE3, CD56	α-FP, β-HCG, h-caldesmon, calponin, CD31, CD99, CD117, CgA, desmin, DOG1, GFAP, HMB45, MelanA, S100, STAT6, Syn
Giovanni Centonze [10]	Not mentioned (but IHC showed an extensive positivity for GLI in nucleus and cytoplasm)	EMA ^e^, CAM5.2 ^e^, AE1/AE3 ^e^, PCK ^e^, LMWCK ^e^, CK7 ^e^, CK19 ^e^, vimentin ^s^, CD10 ^s^, GLI	SMA, CD117, DOG1, TLE1, CD34, CD99, inhibin, SMA, CK20, CK5/6, CDX-2, S100, p63, TTF1, calretinin, Syn, CgA, PDGFRA, p16, estrogen and progesterone receptor
Omar Toumi [11]	Not mentioned	Vimentin, CD99, CD10	Cytokeratin, CgA, Syn, C-kit
Rondell P Graham [4]	*MALAT1-GLI1* fusion	AE1/AE3 ^e^, patchy SMA ^s^	CgA, Syn, KIT, DOG1, desmin, S100, melan-A, SOX10, TLE-1, CD99, keratin5/6
	*MALAT1-GLI1* fusion	Patchy OSCAR ^e^, vimentin ^s^, Ki-67 (approximately 10%)	KRT 34βE12, KRT7, KRT 20, CDX2, CgA, Syn, CD34, CD99, KIT, DOG1, calretinin, WT1, SMA, desmin, EMA, MOC31, melan-A, HMB-45, pCEA
Ma Yangyang [12]	SS18 gene rearrangement was not detected	Vimentin ^s^, CD10 ^s^, CD56 ^s^, PCK ^e^ (AE1/AE3 and CAM5.2), LMWCK ^e^, Ki 67(1% in the most area and 40% in focal areas)	CK7, CK5/6, CK19, CK20, HMWCK, S100, EMA, SMA, desmin, caldesmon, CD34, c-KIT (CD117), PLAP, Syn, CgA, NSE, HMB45, A103, calretinin, ALK, P63, DOG1, CD34, CD99, inhibin, CDX2
Teresa Fernandes [13]	Not mentioned	Vimentin ^s^, PCK ^e^ (AE1/AE3 and CAM5.2), CD56, CD10	C-KIT, DOG-1, CD34, S100, calretinin, CgA, Syn, desmin
Elizabeth A. Wey [2]	*MALAT1-GLI1* fusion	PCK (AE1/AE3/CAM5.2) ^e^, LMWCK ^e^, CK7 ^e^, vimentin ^s^, CD56, CD10, c-KIT ^e^, NSE ^e^, Ki-67 (10%)	CgA, Syn, CEA, TTF-1, PLAP, CD30, AFP, HCG, CK20, calretinin, CDX2, desmin, EMA, inhibin, p63, S100, SMA
Dong Hoon Shin [14]	*MALAT1-GLI1* fusion. C-KIT mutational analysis of exons 9, 11, 13 and 17 showed no abnormality.	PCK (AE1/AE3) ^e^, LMWCK ^e^, EMA ^e^, c-KIT ^e^, CD56, vimentin ^s^, CD10 ^s^	CD34, CEA, CgA, calretinin, desmin, inhibin, NSE, p63, Syn, SMA
Markku Miettinen [3]	None of them demonstrated SS18 gene rearrangement.	Vimentin ^s^, CD10 ^s^, AE1/AE3 ^e^, keratin 18 ^e^, focally keratin 7 ^e^	CD34, CD99, estrogen receptor, KIT, calretinin, keratins5/6,20, CD117, CDX2, CgA, CK5/6, CK20, desmin, EMA, SMA, p63, S100, Syn, TTF1

Notes: IHC (+) refers to immunohistochemistry positive, IHC (−) refers to immunohistochemistry positive. PCK for pancytokeratin, Syn for synaptophysin, CgA for chromogranin A, SMA for smooth muscle actin, SDHB for succinodehydrogenase B, MNF for myocyte nuclear factor, HMB45 for human melanoma black 45, WT1 for Wilms tumor gene-1, FP for fetoprotein, HCG for human chorionic gonadotropin, GFAP for glial fibrillary acidic protein, EMA for epithelial membrane antigen, LMWCK for low-molecular-weight cytokeratin, TLE1 for rransducin-like enhancer 1, TTF1 for thyroid transcription factor 1, PDGFRA for platelet derived growth factor receptor alpha, c-KIT also known as CD117, OSCAR for osteoclast-associated receptor, CDX2 for caudal type homeobox 2, CEA for carcinoma embryonic antigen, HMWCK for high-molecular-weight-cytokeratin, PLAP for placental alkaline phosphatase, NSE for neuron specific enolase, ALK for anaplastic lymphoma kinase, AFP for alpha-fetoprotein, ^e^ refers to only in the epithelial component, ^s^ refers to only in the spindle cell component.

**Table 3:** Treatment choice and related follow-up information of reported cases.

Author	Gastroblastoma			Metastasis	Treatment	Follow-Up Months	Recurrence
	Size (cm)	Location	Infiltration				
Selene C. Koo [6]	6.3	Gastric fundus	Centered on the gastric muscularis propria without subserosal extension	No	Partial gastrectomy	23	No
Long Weiguo [7]	5.0 × 6.0	Great curvature near gastric antrum	Into gastric muscularis propria	No	Tumorectomy	14	No
Diogo Nogueira Pinto [8]	2.27 × 2.18	Great curvature near gastric antrum	Centered in the muscular layer	No	Laparoscopic atypical gastrectomy	18	No
Federica Castri [9]	3, 1.1 and 0.5	Gastric antrum	Submucosa	No	Partial gastrectomy	Not mentioned	Yes ^1^
Giovanni Centonze [10]	5.3	Gastric antrum	Transmural (originated from the muscularis propria with an endoluminal growth)	No	Distal gastrectomy by laparoscopy	100	No
Omar Toumi [11]	7 × 4 × 4	Near the gastric cardia	Transmural with encroachment of the splenic hilum	Yes ^2^	Atypical partial gastrectomy with splenectomy	6	Yes ^3^
Rondell P Graham [4]	Not mentioned	Not mentioned	Not mentioned	No	Resection (not specific described)	12	No
	4.0	Not mentioned	Not mentioned	Yes (liver)	Not mentioned (the patient was diagnosed by needle biopsy)	NA	NA
Ma Yangyang [12]	4.5 × 2.5 × 2.5	In the gastric antrum near the lesser curvature	Transmural	No	Subtotal gastrectomy and gastroduodenostomy	8	No
Teresa Fernandes [13]	10.5 in its largest dimension	Gastric antrum	Centered in the muscular layer	No	Resection of the tumor and partial distal gastrectomy with 15 lymph nodes resected	20	No
Elizabeth A. Wey [2]	3.8 × 3.3 × 2.5	Gastric antrum	Transmural (involved the lamina propria, dissected through the muscularis propria into the subserosa)	Yes ^4^	6 weeks of chemotherapy with no response, and a partial gastrectomy	3	No
Dong Hoon Shin [14]	9.0 × 6.5	Gastric antrum	Centered in the muscularis propria and protruded towards the subserosa	No	Resection of the tumor and a segmental resection of gastric antrum and pylorus during explorative laparotomy.	93	No
Markku Miettinen [3]	5 × 4 × 2.5	Greater curvature of the gastric body	One tumor was transmural, and 2 spanned from superficial muscularis propria into the subserosa.	No	Subtotal gastrectomy	42	No
	6 × 4 × 3.5	Greater curvature of the gastric body		No	Partial gastrectomy	60	No
	15 × 12	Gastric antrum		No	Partial gastrectomy	60	No

Notes: Yes ^1^ refers to that the present neoplasm is the relapse of the disease first presented at his age of 74 which was thought to be paraganglioma at that time, a 90 mm gastric wall tumor with haemorragic pseudocystic degeneration. Yes ^2^ means that there were two lymph nodes at the splenic hilum. Yes ^3^ refers to that after six months of follow-up, the patient developed loco-regional recurrence in the retro-gastric area for which surgical debulking was performed, and she died one month after debulking due to massive pulmonary embolism. Yes ^4^ means that there were peritoneal studding, liver metastases, drop metastases to the pelvis with tumor adherent to the bladder and lymph nodes metastases. Rondell P Graham reported 4 cases, we only listed 2 cases in his part and the other two cases were previously reported by Elizabeth A. Wey (as case 1 in Rondell P Graham’s article) and Dong Hoon Shin (as case 3 in Rondell P Graham’s article). Abbreviations: NA, not available.

**Table 4:** Differential Diagnosis.

Category	More Common in	B/M	Located in	Endoscopy	Size	No.	Morphology	IHC	Mutation
Gastroblastoma	no apparent gender preference, mainly in young adults	M potential	Most seen in gastric antrum	Hypoechoic mass in EUS	0.5–10.5 cm	Single	Biphasic morphology. Epithelioid component contains cords, nests, sheets and tubules of neoplastic cells with vesicular nuclei and small nucleoli. Spindle cell component contains sheets and fascicles of cells with pale eosinophilic cytoplasm, ovoid nuclei with vesicular chromatin and small nucleoli.	Positive for Vimentin, CD10, CD56, PCK.	*MALAT1-GLI1* fusion is the most relevant mutation.
Granular cell tumor	In female and adult	B	Esophagus, colorectum, anus, stomach, appendix and small bowel	As small polyps or plaques	<1 cm	Solitary, sporadic	Composed of sheets and nests of large, polygonal cells with small, hyperchromatic nuclei (with evenly dispersed chromatin) and granular eosinophilic cytoplasm.	Diffusely express SOX10, S-100 protein, CD68, NKI-C3, and overexpress TFE3.	In ATP6AP1, ATP6AP2 and ATP6V0C
Plexiform fibromyxoma	No apparent gender preference, during 40–50 years old	B	Gastric antrum and pylorus, duodenum, jejunum, gallbladder and mediastinum	Appears as a tan/pink, rubbery mass and is centered in the muscularis propria.	0.8–17.0 cm	Single	It exhibits multinodular or plexiform growth of spindly to stellate neoplastic cells in a myxoid stroma. Neoplastic cells are bland, with ovoid to tapering nuclei and delicate eosinophilic cytoplasm. Mitotic figures are rarely seen.	Positive for SMA, vimentin and desmin, negative for ALK, CD34, CD117, KIT, ANO1 (DOG1), EMA and S-100 protein	*MALAT1-GLI1* gene fusions, *GLI1* amplification and *PTCH1* deletions
GIST (gastrointestinal stromal tumors)	No apparent gender preference, in middle-aged adults	M with a metastatic potential	Stomach and other GI tract (small and large intestines)	Hypoechoic mass in EUS which situated within the muscularis propria and may extend to the subserosa.	0.2–30 mm or larger	Sporadic	With various cytologic and growth patterns, the lesion was composed of fascicular or sheet-like growth of spindle cells or of admixed epithelioid and spindle cells.	KIT positive, CD117, DOG1	KIT*

Notes: B/M represents Benign/malignant. No. stands for number and IHC means immunohistochemistry. KIT* refers to KIT (tyrosine kinase receptor gene). Alternate PDGFRA (platelet derived growth factor receptor alpha) kinase mutations were also found in a subset of KIT wild-type GISTs. Other mutations including SDH (succinate dehydrogenase) deficiency, bi-allelic NF1 inactivation and BRAF V600E mutation as well as EGFR mutations and FGFR1, NTRK365 and ALK gene fusions.

## Data Availability

Not applicable.

## References

[1] Papke D.J., Hornick J.L. (2021). Recent developments in gastroesophageal mesenchymal tumours. Histopathology.

[2] Wey E.A., Britton A.J., Sferra J.J., Kasunic T., Pepe L.R., Appelman H.D. (2012). Gastroblastoma in a 28-year-old man with nodal metastasis: Proof of the malignant potential. Arch. Pathol. Lab. Med..

[3] Miettinen M., Dow N., Lasota J., Sobin L.H. (2009). A distinctive novel epitheliomesenchymal biphasic tumor of the stomach in young adults (“gastroblastoma”): A series of 3 cases. Am. J. Surg. Pathol..

[4] Graham R.P., Nair A.A., Davila J.I., Jin L., Jen J., Sukov W.R., Wu T.T., Appelman H.D., Torres-Mora J., Perry K.D. (2017). Gastroblastoma harbors a recurrent somatic *MALAT1-GLI1* fusion gene. Mod. Pathol..

[5] Chen C., Lu J., Wu H. (2022). Case Report: Submucosal gastroblastoma with a novel *PTCH1::GLI2* gene fusion in a 58-year-old man. Front. Oncol..

[6] Koo S.C., LaHaye S., Kovari B.P., Schieffer K.M., Ranalli M.A., Aldrink J.H., Michalsky M.P., Colace S., Miller K.E., Bedrosian T.A. (2021). Gastroblastoma with a novel EWSR1-CTBP1 fusion presenting in adolescence. Genes Chromosomes Cancer.

[7] Long W.G., Zhuang Y., Li M., Zheng F., Zhong A.J., Wang D.Q., Wu J.N. (2020). Gastroblastoma: Report of a case. Zhonghua Bing Li Xue Za Zhi.

[8] Pinto D.N., Ventura J., Gomes D., Brito T., Leite M., Monteiro C., Matos C., Fazeres F., Lopes L., Midões A. (2019). Gastroblastoma described in adult patient. Int. J. Case Rep. Images.

[9] Castri F., Ravegnini G., Lodoli C., Fiorentino V., Abatini C., Giustiniani M.C., Angelini S., Ricci R. (2019). Gastroblastoma in old age. Histopathology.

[10] Centonze G., Mangogna A., Salviato T., Belmonte B., Cattaneo L., Monica M.A.T., Garzone G., Brambilla C., Pellegrinelli A., Melotti F. (2019). Gastroblastoma in Adulthood-A Rarity among Rare Cancers-A Case Report and Review of the Literature. Case Rep. Pathol..

[11] Toumi O., Ammar H., Korbi I., Ayed M., Gupta R., Nasr M., Salem R., Hadhri R., Zayed S., Noomen F. (2017). Gastroblastoma, a biphasic neoplasm of stomach: A case report. Int. J. Surg. Case Rep..

[12] Ma Y., Zheng J., Zhu H., Dong K., Zheng S., Xiao X., Chen L. (2014). Gastroblastoma in a 12-year-old Chinese boy. Int. J. Clin. Exp. Pathol..

[13] Fernandes T., Silva R., Devesa V., Lopes J.M., Carneiro F., Viamonte B. (2014). AIRP best cases in radiologic-pathologic correlation: Gastroblastoma: A rare biphasic gastric tumor. Radiographics.

[14] Shin D.H., Lee J.H., Kang H.J., Choi K.U., Kim J.Y., Park D.Y., Lee C.H., Sol M.Y., Park J.H., Kim H.Y. (2010). Novel epitheliomesenchymal biphasic stomach tumour (gastroblastoma) in a 9-year-old: Morphological, ultrastructural and immunohistochemical findings. J. Clin. Pathol..

[15] Prall O.W.J., McEvoy C.R.E., Byrne D.J., Iravani A., Browning J., Choong D.Y., Yellapu B., O’Haire S., Smith K., Luen S.J. (2020). A Malignant Neoplasm From the Jejunum With a *MALAT1-GLI1* Fusion and 26-Year Survival History. Int. J. Surg. Pathol..

[16] Antonescu C.R., Agaram N.P., Sung Y.S., Zhang L., Swanson D., Dickson B.C. (2018). A Distinct Malignant Epithelioid Neoplasm With GLI1 Gene Rearrangements, Frequent S100 Protein Expression, and Metastatic Potential: Expanding the Spectrum of Pathologic Entities With ACTB/MALAT1/PTCH1-GLI1 Fusions. Am. J. Surg. Pathol..

[17] Chetty R. (2020). Gene of the month: GLI-1. J. Clin. Pathol..

[18] Cohen M., Kicheva A., Ribeiro A., Blassberg R., Page K.M., Barnes C.P., Briscoe J. (2015). Ptch1 and Gli regulate Shh signalling dynamics via multiple mechanisms. Nat. Commun..

[19] Pietrobono S., Gagliardi S., Stecca B. (2019). Non-canonical Hedgehog Signaling Pathway in Cancer: Activation of GLI Transcription Factors Beyond Smoothened. Front. Genet..

[20] Kim S.H., Da Cruz Paula A., Basili T., Dopeso H., Bi R., Pareja F., da Silva E.M., Gularte-Merida R., Sun Z., Fujisawa S. (2020). Identification of recurrent *FHL2-GLI2* oncogenic fusion in sclerosing stromal tumors of the ovary. Nat. Commun..

[21] Punjabi L.S., Goh C.H.R., Sittampalam K. (2022). Expanding the spectrum of GLI1-altered mesenchymal tumors-A high-grade uterine sarcoma harboring a novel PAMR1::GLI1 fusion and literature review of GLI1-altered mesenchymal neoplasms of the gynecologic tract. Genes Chromosomes Cancer.

[22] Ambrosio M., Virgilio A., Raffone A., Arena A., Raimondo D., Alletto A., Seracchioli R., Casadio P. (2022). Malignant epithelioid neoplasm of the ileum with ACTB-GLI1 fusion mimicking an adnexal mass. BMC Womens Health.

